# Fatty acid extracts from *Lucilia sericata *larvae promote murine cutaneous wound healing by angiogenic activity

**DOI:** 10.1186/1476-511X-9-24

**Published:** 2010-03-08

**Authors:** Zhen Zhang, Shouyu Wang, Yunpeng Diao, Jianing Zhang, Decheng Lv

**Affiliations:** 1 Department of Orthopedic Surgery, First Affiliated Hospital, Dalian Medical University, Dalian, Liaoning Province, China; 2 Department of Pharmacy, Dalian Medical University, Dalian, Liaoning Provinc, PR China; 3 Department of Biochemistry, Institute of Glycobiology, Dalian Medical University, Dalian, Liaoning Province, China

## Abstract

**Background:**

fatty acids are considered to be effective components to promote wound healing and *Lucilia sericata *larvae are applied clinically to treat intractable wounds. We aimed to investigat the effect of fatty acid extracts from dried *Lucilia sericata *larvae on murine cutaneuous wound healing as well as angiogenesis.

**Results:**

On day 7 and 10 after murine acute excision wounds creation, the percent wound contraction of fatty acid extracts group was higher than that of vaseline group. On day 3, 7 and 10 after wounds creation, the wound healing quality of fatty acid extracts group was better than that of vaseline group on terms of granulation formation and collagen organization. On day 3 after wounds creation, the micro vessel density and vascular endothelial growth factor expression of fatty acid extracts group were higher than that of vaseline group. Component analysis of the fatty acid extracts by gas chromatography-mass spectrometry showed there were 10 kinds of fatty acids in total and the ratio of saturated fatty acid, monounsaturated fatty acid and polyunsaturated fatty acid (PUFA) was: 20.57%:60.32%:19.11%.

**Conclusions:**

Fatty acid extracts from dried Lucilia sericata larvae, four fifths of which are unsaturated fatty acids, can promote murine cutaneous wound healing probably resulting from the powerful angiogenic activity of the extracts.

## Background

Fatty acids, as a family of bioactive moleculars, participate in many metabolic processes. The deficiency of fatty acids may result in diseases, vice versa supplementing fatty acids can treat diseases. Since a kind of disease is a result of multiple etiological factors, it is difficult to get a satisfactory therapeutic outcome using a single compound as drug. Therefore more and more doctors focus on ethnopharmacolgical theory, which records folk plants or animals containing many effective components. The therapeutic utilization of *Lucilia sericata *larvae commonly called maggot therapy for wound healing can date back to the beginning of civilization [1]. According to traditional Chinese medicine principle and the book *Compendium of Materia Medica *records, *Lucilia sericata *larvae which has been given the name of "*WuGuChong*" is salty in taste, cold in nature and non-toxic. It is mainly produced in Guangdong Province in China and local people use "*WuGuChong*" to treat superficial purulent diseases such as furuncle or carbuncle. Recently, maggot therapy has been widely used to treat various refractory wounds including diabetes foot and venous ulcers [2-5]. The therapeutic action comprises biological debridement and enhancement of wound healing. The mechanisms of debriding effect of maggots were due to their mechanical wriggling and excretions/secretions degrading extracellular matrix [6,7]. However, there are no *in vivo *reports regarding wound healing mechanisms of maggot therapy. In addition, complications such as larvae loss, persistent pain or severe systemic infection [8] induced by live maggot limits the clinical use of maggot therapy, so the theory and practice of new delivery method is urgent.

Cutaneous wound healing is a complex process, consisting of an orderly progression of inflammation, angiogenesis, collagen deposition, re-epithlization and tissue remolding [9]. The purpose of repairing events is to resist pathogens invasion, establish the integrity of the damaged tissue and reconstruct physiological function of skin [10]. This kind of process also involves a variety of systemic and local factors such as chemokines [11], growth factors and neuropeptides [12] that exert their respective function. It is almost certain that neovascularization composed of angiogenesis and vasculogenesis is one of the most important events in the healing process and is a response to tissue injury in order to restore vascular perfusion to provide nutrition and oxygen to the repairing tissue [13,14]. When angiogenesis is initiated by vasodilation, the activated endothelial cells migrate and proliferate to form new capillaries by sprouting or splitting from their vessel of origin [15]. Vasculogenesis is a *de novo *process that also play an important role in wound healing through incorporation, differentiation, migration and proliferation of bone marrow-derived endothelial progenitor cells that contribute to existing and new vascular channels[16]. Vascular endothelial growth factor A(VEGFA), a mitogen for vascular endothelium, is expressed not only by skin resident cells but also by inflammatory cells when healing process happens. VEGFA is considered to be a potent and crucial angiogenic stimulator and promotes all steps in the cascade process of angiogenesis by binding to the extracellular domain of its receptor to activate the downstream proteins after the dimerization and autophosphorylation of the intracellular receptor tyrosine kinases [17].

The previous study in our laboratory demonstrated that 1) *Lucilia sericata *larvae excretions/secretions induced human microvascular endothelial cell migration through v-akt murine thymoma viral oncogene homolog 1(AKT1) pathway [18]; 2) the homogenate product from *Lucilia sericata *larvae enhanced wound healing by promoting wound nerve regeneration. In present study, we utilized fatty acid extracts from dried *Lucilia sericata *larvae to investigate the effect of the extracts on wound healing, angiogenesis and VEGFA expression in murine wound model.

## Methods

### Drugs and Extraction

*Lucilia sericata *larvae which refer to the dried bodies of *Lucilia sericata., Calliphoridae *from Guangdong Province of China were purchased in a traditional Chinese medicine market in Anguo city, and identified by YunPeng Diao professor in Dalian Medical University. A voucher specimen is kept and the number is QU20090302. The bodies were grinded into powder, suspended in AcOEt and extracted at 50°C water bath for about 3 hours. Filter liquor was adding water and titrated by 0.5 mol/L NaOH-EtOH till titration end-point. By separating water-organic phase, the water phase was obtained, acidulated by 1.0 mol/L HCl and extracted by AcOEt. Fatty acid extracts were obtained by reduced pressure concentration and chilling drying process.

### Animals

Healthy, male, Sprague-Dawley rats, weighing 200-220 g, were supplied by the Animal Experimental Center of Dalian Medical University and housed one per cage for a week before study in a room with controlled environment (12-h light/dark cycle, 23 ± 2°C and relative humidity 70%). They were also given free access to standard laboratory diet and water. All experimental procedures were approved by the Animal Research Ethics Committee of Dalian Medical University, Dalian, China.

### Acute excision wounds creation and treatment

The animals were anesthetized by an intraperitoneal injection of 10% chloral hydrate (0.3 ml/100 g), and the dorsal surface of the rat was shaved and the underlying skin was cleaned with povidone iodine. An acute 1.5 cm diameter circle full thickness excision wound was created by using a scalpel blade on the back of each animal. All the animals were divided into three groups randomly (30 animals in each group). Animals in groupIwere treated by fatty acid extracts of dried *Lucilia sericata *larvae 0.1 g per wound. Animals in groupIIwere treated by vaseline ointment 0.1 g per wound and served as negative control. Animals in groupIII were treated by *JingWanHong *ointment (a kind of traditional Chinese patent medicine to treat wounds, Tian Jin Darentang Daer Pharmacy Co,. Ltd, China) 0.1 g per wound and served as positive control. All drugs were applied topically every other day until the wounds completely healed.

### Wound measurement

On day 1, 3, 7, 10, and 14 after wound creation, 6 animals in each group were sacrificed randomly. The wound diameter was measured and the area (mm^2^) within the boundary was calculated planimetrically. The percentage wound contraction was determined using the following formula: percent wound contraction = (original wound area- unhealed area)/original wound area × 100%. Meanwhile the wound tissues were excised to analyze by histology, immunohistochemistry, reverse transcriptase polymerase chain reaction (RT-PCR) and Western-blot.

### Histological examination of excised tissue

The excised wound tissue was fixed in 10% neutral buffered formalin, dehydrated in graded ethanol, cleared in xylene, and embedded in paraffin. Five-micron-thick sections, including the epidermis, the dermis, and the subcutaneous panniculus carnosus muscle, were mounted on glass slides, dewaxed, rehydrated to distilled water, and stained with hematoxylin and eosin(HE) or Masson's Trichrome. All subsequent analyses were performed by an experienced pathologist without knowledge of the previous treatment. A five-tiered grading system based on degree of re-epithelization, granulation tissue formation and collagen organization was adopted to evaluate the historical differences of different samples, as described in Additional file 1[19,20].

### Immunohistochemical analysis of micro vessel density (MVD) and VEGFA expression

The available wound tissue sections were deparaffinized and redehydrated. Then 3% H_2_O_2 _in methanol was used for 10 minutes to block endogenous peroxidase activity. The sections were boiled in 0.01 mol/L citric acid for 20 minutes to retrieve the antigen. To prevent nonspecific binding, normal goat serum was applied for at 37°C for 10 minutes, then the sections were reacted with mouse anti-rat CD31 monoclonal antibody (diluted 1:5, Abcam, UK) or mouse anti-rat VEGFA monoclonal antibody (diluted 1:5, Abcam, UK) at 37°C for 1 hour. After washing with PBS, sections were incubated with biotinylated goat anti-mouse antibody (Zhongshan Biology Technology Co., Ltd., China) at 37°C for 30 minutes and then horseradish peroxidase-labeled streptavidin at 37°C for 30 minutes. After 3,3'-diaminobenzidine(DAB)/H_2_O_2 _staining and hematoxylin staining, sections were dehydrated, cleared, and mounted for viewing. For MVD analysis, the sections were examined under 100× magnication to identify the highest vascular density area within the wounds, and five areas of the highest vascular density area were selected for counting under 400× magnication (0.16 mm^2^/field). The number of CD31 positive small vessels was counted under 400× magnication to determine the density of micro vessels. Any brown-staining endothelial cell or endothelial cell cluster that was clearly separated from adjacent micro vessels was considered a single, countable micro vessel. MVD was then expressed as number of counted micro vessels per mm^2^. The average MVD of five areas selected was considered as MVD of this sample. For VEGFA analysis, the sections were firstly examined under 100× magnication to identify the highest positive expression within the wound, and five areas of the highest expression were then selected for evaluating under 400× magnication. The images were analyzed by Image-pro-plus 6.0 software (Media Cybernetics, USA) to calculate the area, density mean and integrated optical density (IOD) of positive expression. The average result of the five areas was recorded as the statistic data of this sample.

### Analysis of VEGFA mRNA expression by RT-PCR

Total RNA was isolated from homogenized excised tissues using Trizol (Invitrogen, USA), and cDNA was synthesized using RT-PCR kit (TaKaRa, Japan) according to the manufacturer's protocol. The oligonucleotide sequences of the primers of VEGFA were 5'-TGCACCCACGACAGAAGGGGA-3' for sense and 5'-TCACCGCCTTGGCTTGTCACAT-3' for antisense; while the sequences of primer of GAPDH used as a control were 5'-GGCCGTGAAGTCGTCAGAAC-3' for sense and 5'-GCCACGATGCCCAGGAA-3' for antisense. PCR conditions were as follows: denaturation at 95°C for 3 minutes, and then 30 cycles of denaturation for 20 seconds at 94°C, annealing for 30 seconds at 55°C and extension for 30 seconds at 72°C. The PCR products were separated with electrophoresis using 1.0% agarose gel, and photographed under ultraviolet radiation light. Band intensities were measured using Gel-Pro Analyzer 6.0 software (Media Cybernetics, USA) and were normalized to those for GAPDH.

### Analysis of VEGFA protein expression by Western-blot

Excised tissues were homogenized with lysis buffer and centrifuged. The supernatant lysate were mixed with 2× sodium dodecyl sulfate (SDS) sample buffer (0.5 M Tris-HCL pH 6.8, 10% SDS, 5%glycerol and 5% β-mercaptoethanol). Equal amounts of denatured proteins were resolved by 10% SDS-PAGE and then transferred to nitrocellulose membranes (Pall Corporation, USA). After blocking for 1 hours with phosphate-buffered saline containing 0.1% Tween 20 and 5% powdered skim milk at room temperature, the membranes were incubated with mouse anti-rat VEGFA monoclonal antibody (diluted 1:1000, Abcam, UK) 37°C for 4 hours in 5% powdered skim milk buffer, washed thrice with phosphate-buffered saline with 0.1% Tween 20 and then incubated with secondary antibody anti-mouse HRP (diluted 1:5000, Santa Cruz Biotech, USA). GAPDH antibody (diluted 1:500, Santa Cruz Biotech, USA) was used as a control. All bands were detected using ECL Western-blot kit (Amersham Biosciences, UK). Protein band intensities were determined using Gel-Pro Analyzer 6.0 software (Media Cybernetics, USA) and were normalized to those for GAPDH.

### Component analysis of fatty acid extracts

Some fatty acid extracts were taken into 25 ml tube, 4 ml benzene-petroleum ether (V:V = 1:1) and 4 ml KOH-MEOH (0.4 mol/L) were added, respectively. After vortexing, the tubes were heated for 30 ± 1 minutes at 50° ± 1°C water bath, and 10 ml deionized water were added. The supernatant was as the sample for gas chromatography-mass spectrometry. A HP-5 MS capillary column (28 m × 0.25 mm × 0.25 μm) with helium as carrier gas (1.0 ml/min) was used for the gas chromatographic separation. The injection mode was split (split ratio: 20:1), injection volume was 2 μL, and temperature of vaporization chamber was 250°C. The following temperature program was applied: 1 min at 100°C, then 8°C/min up to 200°C, then 3 min at 200°C, 1°C/min up to 220°C and 3 min at 220, last 6°C/min up to 280°C and 2 min at 280°C. Mass detector was EI-Detection (70 eV).

### Statistical analysis

Every 6 of 30 animals per group were sacrificed randomly at 5 different time points. All the presented data was expressed as mean ± standard deviation, and the statistical significances among three groups were analyzed by one-way ANOVA, and then differences among means were analyzed with Fisher's least significant difference t-test using the SPSS version 13.0 software (*SPSS Inc., USA*). *P *value less than 0.05 was considered as statistically significant.

## Results

### The percent wound contraction

On day 1 and 3 after wound creation, the percent wound contraction was no significant difference among three groups. On day 7 and 10, the percent wound contraction of study group and positive control group was higher, as compared with negative control group(P < 0.05)(Additional file 2). Wounds in three groups healed until day 14.

### Histological examination of excised tissues (HE and Masson's Trichrome)

**Figure 1:**
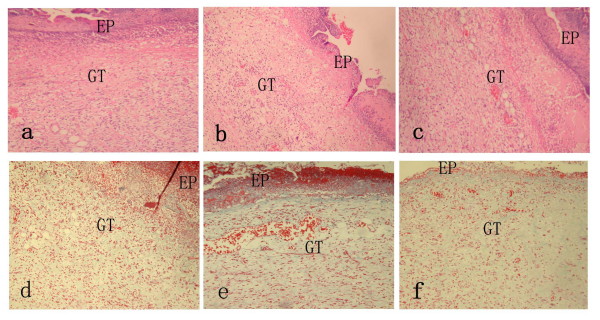
**Histological compare in the character of wound healing on day 3 (100× magnification)**. a(HE), d(Masson Trichrome) in study group and c(HE), f(Masson Trichrome) in positive control group showed the thick granulation tissue layer with robust newly-formed vessels as well as plenty of inflammatory and repair cells. b(HE), e(Masson Trichrome) in negative control group showed massive necrotic substances and a thin granulation tissue layer. EP, epithelium; GT, granulation tissue.

On day 1, the re-epithelialization, granulation and collagen deposition were no significant difference among three groups with a thin and incomplete re- epithelialization, abundant fibrinous exudation, few vessels and trace collagen. On day 3, wounds of both study group and positive control group displayed a more accumulation of granulation tissue with a high degree of newly-formed micro-vessels and numerous inflammatory cells and fibroblast (Fig. 1). The mean values of parameters related to wound healing in study group and positive control group were higher compared to negative group (*P *< 0.05) except re-epithelialization (Additional file 3). On day 7 and day 10, wounds of all three groups demonstrated a continuous epithelial line covering the whole wound bed. Moreover, in study group and positive control group, the granulation was matured showing capillary vertically oriented, robust fusiform fibrocytes and moderate well-arranged collagen. In contrast, in negative control group, the granulation tissue was un-matured with capillary poorly-organized, many fibroblast and slight collagen formation. On day 14, all the wounds healed, and there was no significant difference among three groups according to histological examination.

### Immunohistochemical examination of MVD and VEGFA expression

**Figure 2:**
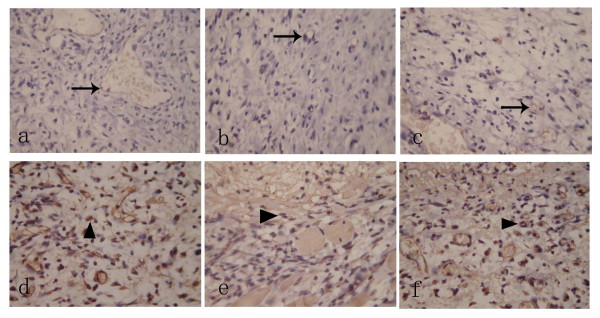
**Immunohistochemical examination of MVD and VEGFA expression on day 3 (400× magnification)**. There was higher MVD (Black arrow) in study group (a) and positive control group (c) as compared with negative group (b). VEGFA was mainly expressed in neutrophils, macrophages, endothelial cells and fibroblasts within granulation tissue (Black triangle). Compared with negative control group (e), the study group (d) and positive control group (f) expressed more with respect to VEGFA.

On day 3, the MVD, area and IOD of VEGFA expression were at the climax among all three groups along the whole healing process (Fig. 2). While such mentioned parameters of study group and positive group were higher than that of negative group (*P *< 0.05). Additionally, compared with positive control group, the MVD and VEGFA expression of study group were higher (*P *< 0.05)(Additional file 4 and Additional file 5). There was no significant difference at other time points.

### VEGFA mRNA expression by RT-PCR and VEGFA protein expression by Western-blot

**Figure 3:**
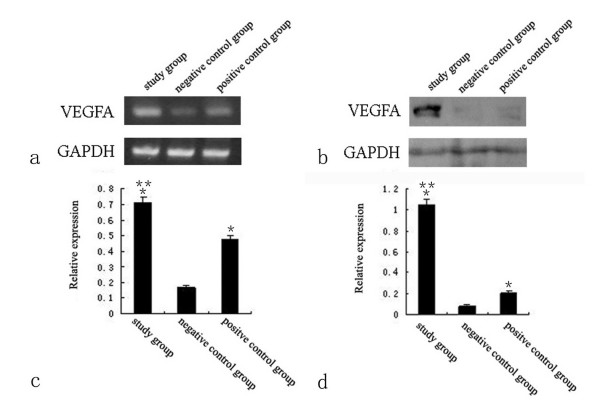
**VEGFA mRNA and protein expression on day 3**. VEGFA mRNA expression revealed by RT-PCR (a) and VEGFA protein expression determined by western-blot (b) and its relative expression (c and d) showed the expression of study group was the highest and the expression of negative control group was the lowest(*, compared with negative control group; **, compared with positive control group).

On day 3, the VEGFA mRNA expression in wounds determined by RT-PCR was seen in all three groups (Fig. 3a). Comparatively, the expression of both study group and positive control group was higher than that of negative control group (*P *< 0.05). Furthermore, the expression of study group was higher than that of positive control group (*P *< 0.05)(Fig. 3c). Similarly, the VEGFA protein expression in wounds was detected in all three groups (Fig. 3b), and the expression was up-regulated in both study group and positive control group as compared with negative control group(*P *< 0.05). Moreover, the VEGFA protein was expressed more in study group as compared with positive control group (*P *< 0.05)(Fig. 3d). The VEGFA mRNA or protein expression was no significant difference among three groups at other time point.

### Component analysis of the fatty acid extracts by GC-MS

**Figure 4:**
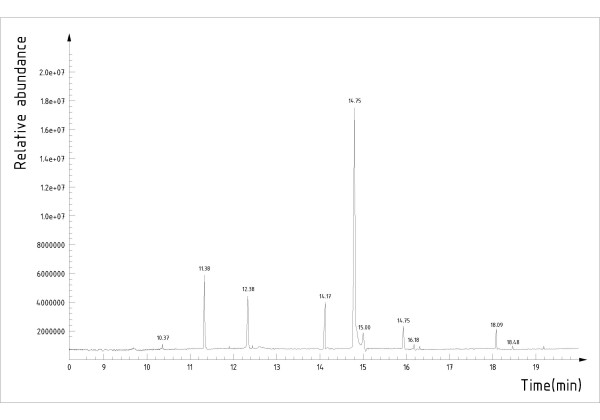
**Total ion chromatography of fatty acids extract from *Lucilia sericata *larvae**. There are ten different peaks representing ten kinds of fatty acids.

The fatty acid methyl esterifications were identified using GC-MS and the relative content of each component was determined by peak area normalization method. There were 10 kinds of fatty acids in total (Fig. 4) and the ratio of saturated fatty acid (SFA), monounsaturated fatty acid (MUFA) and polyunsaturated fatty acid (PUFA) was: 20.57%:60.32%:19.11%(Additional file 6).

## Discussion

For thousands of years, people all over the world have been engaging in searching for effective natural products involved in insects or plants to treat skin injury. In present study, the fatty acid extracts of dried *Lucilia sericata *larvae could up-regulate immunohistochemical, transcriptional and translational level of VEGFA expression and increase the amount of new-formed capillary at inflammatory phase as well as the percent wound contraction at granulation formation phase and scar remolding phase. The enhanced effect of the extracts on angiogenesis and wound healing did not happen simultaneously. Prior to promoting wound healing, the extracts demonstrated a stronger angiogenic effect, even than *JingWangHong*, let alone Vaseline. So we can infer that the fatty acid extracts of dried *Lucilia sericata *larvae promote wound healing characterized by a shorten period of healing time, probably due to their associated powerful angiogenic property. Since neovascularity, an indicator of immature granulation tissue, diminishes gradually as wound mature [10,20], the extracts did not influence MVD and VEGFA expression at late stage of healing process resulting in an acceleration of wound maturity. In addition, according to our observation, after the extracts were applied, the rats did not show any signs of restlessness or scratching wound site, partly suggesting that the extracts did not cause irritation or pain to the animals. However, whether the extracts cause other side effects needs further formal and systemic investigations.

The mechanism of how fatty acid extracts acts on angiogenesis and wound healing may involve multiple pathways. Monounsaturated fatty acid (MUFA) such as oleic acid and polyunsaturated fatty acid (PUFA) such as arachidonic acid affect the function of vessels in several aspects, involved in newly-formed micro-vessels formation and VEGFA expression in the wound healing process [21,22]. Fatty acids like arachidonic acid and arachidonic acid precursors as fundamental constituent of plasma membranes are metabolized by cyclooxygenase (COX), lipoxygenase (LOX) or cytochrome P450 (CYP) enzymes [23] and their metabolites are mediators of several events such as cellular growth, angiogenesis and extracellular matrix synthesis [24], when wound healing happens. Such fatty acids are consumed continuously to produce intracellular messengers in turn mediating a number of biological activities including endothelial cell proliferation [25]. It is reported that PUFA can regulate epithelial cell proliferation *in vitro *[26] and angiogenesis in vivo by ERK, p38 MAPK, or PI3K signal pathway [27]. Healing process will be no doubt disrupted, if no enough unsaturated fatty acids are supplied. In present study, fatty acid extracts, as a kind of nutrient substances, supplemented essential fatty acid for wound healing consumption.

It is well known that the presence of unsaturations makes fatty acids possess an anti-oxidative effect related to reacting with reactive oxygen species (ROS) called peroxidation. At the early stage of healing process, inflammatory cells such as neutrophils and macrophages release a high amount of ROS by an oxygen consuming respiratory burst [28,29]. The ROS play a dual regulative role for wound metabolism. It is reported that low concentration of oxygen free radicals in the wound site can promote angiogenesis by inducing VEGFA expression in keratinocytes [30] and macrophages [31] as well as stimulate collagen production [32]. However at high concentration, oxygen free radicals exhibit obvious tissue damage action [33,34] and induce apoptosis of wound repair cells [35]. In present study, the fatty acid extracts containing plenty of unsaturated fatty acids may maintain ROS at a relatively low concentration[28,29], thus to enhance wound healing by its oxygen free radical scavenging effect. In addition, the decrease of apoptosis of endothelial cells induced by ROS may also contribute to the promotion of angiogenesis. To sum up, the angiogenic activity of fatty acid extracts of dried *Lucilia sericata *larvae is related to its synergistic effects of both proliferation and anti-oxidation on endothelial cells.

## Conclusions

Fatty acid extracts of dried Lucilia sericata larvae, four fifths of which are unsaturated fatty acids, can promote murine cutaneous wound healing probably resulting from the powerful angiogenic activity of the extracts.

## List of Abbreviations

COX: cyclooxygenase; CYP: cytochrome P450; IOD: integrated optical density; GC-MS: gas chromatography-mass spectrometry; LOX: lipoxygenase; MUFA: monounsaturated fatty acid; MVD: Micro vessel density; PUFA: polyunsaturated fatty acid; ROS: reactive oxygen species; RT-PCR: reverse transcriptase polymerase chain reaction; VEGFA: vascular endothelial growth factor A; AKT1: v-akt murine thymoma viral oncogene homolog 1.

## Competing interests

The authors declare that they have no competing interests.

## Authors' contributions

All the authors participated in formulating the hypothesis, executing the work, analyzing the data, writing the manuscript and approved the final version for submission. Besides, ZZ performed the majority of the experiments and DL provided financial supports.

## Supplementary Material

Additional file 1 Score of historical evaluation. Click here for file

Additional file 2 The percent wound contraction at different time point. Click here for file

Additional file 3 Wound healing parameters of histological examination at different time point. Click here for file

Additional file 4 The micro vessels density (no. small vessels/mm^2^) at different time point. Click here for file

Additional file 5 The expression of VEGFA at different time point. Click here for file

Additional file 6 The components of fatty acid extracts of dried *Lucilia sericata *larvae. Click here for file
