# Supplementary material for: Fatty acid extracts from Lucilia sericata larvae promote murine cutaneous wound healing by angiogenic activity
Source: Lipids Health Dis. 2010 Mar 8;9:24. doi: 10.1186/1476-511X-9-24 (PMC2841600; doi:10.1186/1476-511X-9-24)
Supplement: Additional file 1 — Score of historical evaluation. [file 1476-511X-9-24-S1.DOC]

Additional file 1: Score of historical evaluation

| Score | Re-epithelialization | Granulation tissue formation | Collagen organization |
| --- | --- | --- | --- |
| 0 | None | None | None |
| 1 | Migrating | Hypo cellular with few vessels | Trace |
| 2 | Partial stratum corneum | Many vessels and some cells | Slight |
| 3 | Hypertrohic | Many fibroblasts, some fibers | Moderate |
| 4 | Complete and normal | More fibers, few cells | Marked |
