# Supplementary material for: Fatty acid extracts from Lucilia sericata larvae promote murine cutaneous wound healing by angiogenic activity
Source: Lipids Health Dis. 2010 Mar 8;9:24. doi: 10.1186/1476-511X-9-24 (PMC2841600; doi:10.1186/1476-511X-9-24)
Supplement: Additional file 2 — The percent wound contraction at different time point. [file 1476-511X-9-24-S2.DOC]

| Group | Wounds | Original wound area(cm^2^) | Percent wound contract (%) | | | | |
| --- | --- | --- | --- | --- | --- | --- | --- |
|  |  |  | Day 1 | Day 3 | Day 7 | Day 10 | Day 14 |
| Study group | 6 | 1.80±0.24 | 7.23±0.23 | 20.15±2.00 | 69.48±2.06^a^ | 88.41±2.15 ^a^ | 100 |
| Negative control group | 6 | 1.81±0.23 | 7.23±0.19 | 19.31±1.83 | 65.18±1.22 | 80.41±1.78 | 100 |
| Positive control group | 6 | 1.80±0.17 | 7.24±0.20 | 20.21±1.92 | 70.01±2.91 ^a^ | 88.31±3.24 ^a^ | 100 |

Additional file 2 - The percent wound contraction at different time point

Values are mean ± S.D. of six wounds in each group.

^a^*P*<0.05 as compared to control group.
