# Supplementary material for: Fatty acid extracts from Lucilia sericata larvae promote murine cutaneous wound healing by angiogenic activity
Source: Lipids Health Dis. 2010 Mar 8;9:24. doi: 10.1186/1476-511X-9-24 (PMC2841600; doi:10.1186/1476-511X-9-24)
Supplement: Additional file 3 — Wound healing parameters of histological examination at different time point. [file 1476-511X-9-24-S3.DOC]

Additional file 3 - Wound healing parameters of histological examination at different time point

| Group | Day 1 | | |  | Day 3 | | |  | Day 7 | | |  | Day 10 | | |  | Day 14 | | |
| --- | --- | --- | --- | --- | --- | --- | --- | --- | --- | --- | --- | --- | --- | --- | --- | --- | --- | --- | --- |
|  | Re-epithelialization | granulation | collagen |  | Re-epithelialization | granulation | collagen |  | Re-epithelialization | granulation | collagen |  | Re-epithelialization | granulation | collagen |  | Re-epithelialization | granulation | collegen |
| Study group | 1.20 | 1.45 | 0.76 |  | 2.50 | 2.95 ^a^ | 2.71 ^a^ |  | 2.96 | 3.66 ^a^ | 3.65 ^a^ |  | 3.56 | 3.90 ^a^ | 3.82 ^a^ |  | 4 | 4 | 4 |
| negative control group | 1.20 | 1.41 | 0.73 |  | 2.48 | 2.08 | 1.95 |  | 2.93 | 2.70 | 2.89 |  | 3.50 | 3.05 | 3.32 |  | 4 | 3.94 | 3.91 |
| positive control group | 1.21 | 1.43 | 0.74 |  | 2.50 | 2.93 ^a^ | 2.74 ^a^ |  | 2.96 | 3.56 ^a^ | 3.66 ^a^ |  | 3.55 | 3.94 ^a^ | 3.85 ^a^ |  | 4 | 4 | 4 |

Values are mean ± S.D. of six wounds in each group.

^a^*P*<0.05 as compared to control group.
