# Supplementary material for: Fatty acid extracts from Lucilia sericata larvae promote murine cutaneous wound healing by angiogenic activity
Source: Lipids Health Dis. 2010 Mar 8;9:24. doi: 10.1186/1476-511X-9-24 (PMC2841600; doi:10.1186/1476-511X-9-24)
Supplement: Additional file 4 — The micro vessels density (no. small vessels/mm2) at different time point. [file 1476-511X-9-24-S4.DOC]

Additional file 4 - The micro vessels density (no. small vessels/mm^2^) at different time point

| Group | Day 1 | Day 3 | Day 7 | Day 10 | Day 14 |
| --- | --- | --- | --- | --- | --- |
| Study group | 49.36±11.32 | 100.17±28.33 ^ab^ | 64.11±12.93 | 33.34±9.20 | 8.23±1.20 |
| Negative control group | 47.47±11.19 | 76.83±15.07 | 66.34±13.10 | 35.25±13.67 | 8.46±2.11 |
| Positive control group | 48.04±10.57 | 90.42±26.65 ^a^ | 64.34±10.29 | 34.54±10.88 | 8.93±2.91 |

Values are mean ± S.D. of six wounds in each group.

^a^*P*<0.05 as compared to control group.

^b^*P*<0.05 as compared to positive control group.
