# Supplementary material for: Fatty acid extracts from Lucilia sericata larvae promote murine cutaneous wound healing by angiogenic activity
Source: Lipids Health Dis. 2010 Mar 8;9:24. doi: 10.1186/1476-511X-9-24 (PMC2841600; doi:10.1186/1476-511X-9-24)
Supplement: Additional file 5 — The expression of VEGFA at different time point. [file 1476-511X-9-24-S5.DOC]

Additional file 5 - The expression of VEGFA at different time point

| Group | Day 1 | | |  | Day 3 | | |  | Day 7 | | |  | Day 10 | | |  | Day 14 | | |
| --- | --- | --- | --- | --- | --- | --- | --- | --- | --- | --- | --- | --- | --- | --- | --- | --- | --- | --- | --- |
|  | Area(μm2) | Density mean | IOD |  | Area(μm2) | Density mean | IOD |  | Area(μm2) | Density mean | IOD |  | Area(μm2) | Density mean | IOD |  | Area(μm2) | Density mean | IOD |
| Study group | 61.39±19.12 | 0.38±0.01 | 23.05±6.87 |  | 251.31±34.23 ^ab^ | 0.41±0.01 | 106.22±15.92 ^ab^ |  | 100.92±22.94 | 0.41±0.01 | 43.35±7.83 |  | 34.37±11.28 | 0.37±0.01 | 13.33±2.01 |  | 9.50±2.11 | 0.35±0.01 | 3.33±0.56 |
| Negative control group | 61.81±10.10 | 0.38±0.01 | 22.38±6.01 |  | 132.02±30.21 | 0.40±0.01 | 52.12±11.99 |  | 98.54±20.81 | 0.41±0.01 | 42.65±7.02 |  | 33.65±10.09 | 0.37±0.01 | 12.57±2.22 |  | 9.68±2.02 | 0.35±0.01 | 3.40±0.44 |
| Positive control group | 62.00±15.38 | 0.38±0.01 | 22.65±6.2 |  | 202.53±33.89^a^ | 0.41±0.01 | 85.91±13.90^a^ |  | 98.30±22.37 | 0.41±0.01 | 42.96±7.32 |  | 35.04±13.33 | 0.37±0.01 | 13.67±1.89 |  | 9.32±2.62 | 0.35±0.01 | 3.25±0.39 |

Values are mean ± S.D. of six wounds in each group.

^a^*P*<0.05 as compared to control group.

^b^*P*<0.05 as compared to positive control group.
