# Supplementary material for: Fatty acid extracts from Lucilia sericata larvae promote murine cutaneous wound healing by angiogenic activity
Source: Lipids Health Dis. 2010 Mar 8;9:24. doi: 10.1186/1476-511X-9-24 (PMC2841600; doi:10.1186/1476-511X-9-24)
Supplement: Additional file 6 — The components of fatty acid extracts of dried Lucilia sericata larvae. [file 1476-511X-9-24-S6.DOC]

Additional file 6 - The components of fatty acid extracts of dried *Lucilia sericata* larvae

| No. | Retention time(min) | Compound | Molecular formula | Relative content(％) |
| --- | --- | --- | --- | --- |
| 1 | 10.37 | Tetradecanoic acid | C_14_H_28_O_2_ | 1.85 |
| 2 | 11.38 | 9-hexadecenoic acid | C_16_H_30_O_2_ | 17.45 |
| 3 | 12.38 | Palmic acid | C_16_H_32_O_2_ | 15.02 |
| 4 | 14.17 | 9,12-linoleic acid | C_18_H_32_O_2_ | 11.81 |
| 5 | 14.75 | 9-oleic acid | C_18_H_34_O_2_ | 36.53 |
| 6 | 15.00 | 10-oleic acid | C_18_H_34_O_2_ | 6.34 |
| 7 | 15.88 | octadecoic acid | C_18_H_36_O_2_ | 3.70 |
| 8 | 16.18 | 7,10-oleic acid | C_18_H_32_O_2_ | 2.21 |
| 9 | 18.09 | 5,8,11,14-arachidonic acid | C_20_H_32_O_2_ | 4.55 |
| 10 | 18.48 | 7,10,13-eicosatrienoic acid | C_20_H_34_O_2_ | 0.54 |
